# 高容量双酚A分子印迹聚合物的可控合成及其在环境水样检测中的应用

**DOI:** 10.3724/SP.J.1123.2025.04029

**Published:** 2026-01-08

**Authors:** Yun CHENG, Yule LIN, Miaomiao TIAN

**Affiliations:** 哈尔滨师范大学化学化工学院，黑龙江 哈尔滨 150025; College of Chemistry and Chemical Engineering，Harbin Normal University，Harbin 150025，China

**Keywords:** 分子印迹聚合物, 双酚A, 高效液相色谱, 选择性吸附, molecularly imprinted polymers （MIPs）, bisphenol A （BPA）, high performance liquid chromatography （HPLC）, selective adsorption

## Abstract

针对环境水样中双酚A（BPA）迁移污染问题，本研究构建了Co/Ni双金属有机框架（MOF）基分子印迹材料（CoNi-MOF-MIPs）。通过盐酸多巴胺（DA）自聚合策略，在双金属MOF表面构筑选择性识别位点，系统优化功能单体（DA）与模板分子（BPA）的配比，其最佳质量比为5∶4。同时，优化了聚合时间及吸附pH等关键参数，最终确定最佳聚合时间为5 h，最佳吸附pH为4.0。扫描电子显微镜（SEM）表征证实材料具有纳米花状结构，该结构能提供较多的吸附位点。材料的吸附动力学符合拟二级模型（*R* ^2^=0.987 9），最大吸附量达39.29 mg/g，印迹因子较高（3.48），且经6次重复利用后仍保持93.2%的吸附效率。结合高效液相色谱构建的检测体系在0.17~40μg/mL范围内呈现良好的线性关系（*R*^2^=0.997 4），对环境水样的加标回收率为80.3%~91.7%（相对标准偏差<1.8%），该方法的检出限为0.05 μg/mL，可实现对环境水样中BPA的高效富集和检测。本工作所开发的BPA表面分子印迹聚合物具有良好的实际应用能力。

## Abstract (en)

Bisphenol A （BPA） is an endocrine-disrupting chemical that mimics estrogen， thereby interfering with human hormonal balance and leading to reproductive abnormalities， developmental disorders， and increased risks of obesity， diabetes， and cancer. Commonly found in plastic products and food packaging materials， BPA can readily leach from packaging under thermal stress or exposure to acidic/alkaline conditions， subsequently migrating into the environment. This results in its widespread presence in surface water， groundwater， and drinking water systems. Prolonged exposure poses significant health threats， while conventional wastewater treatment processes prove inadequate for its complete removal. Therefore， developing an efficient， highly specific， and reusable material for removing BPA from water systems is of substantial practical significance. Molecularly imprinted polymers （MIPs） have emerged as promising candidates for targeted pollutant removal due to their artificially created recognition sites that exhibit both structural and functional complementarity to template molecules. The unique advantages of MIPs， including their exceptional specificity， chemical stability， and reusability， make them particularly suitable for environmental applications. To address the challenge of BPA contamination in water systems， we developed a novel cobalt-nickel bimetallic metal-organic framework-based molecularly imprinted polymer （CoNi-MOF-MIPs） through an innovative surface imprinting approach. The fabrication process involved multiple carefully controlled steps. Initially， a crystalline CoNi-MOF substrate was synthesized as the supporting matrix， providing high surface area and structural stability. Dopamine hydrochloride （DA） was then employed as the functional monomer， which underwent self-polymerization under weakly alkaline conditions to form a polydopamine （PDA） coating while simultaneously immobilizing BPA template molecules through synergistic hydrogen bonding and *π*-*π* interactions. Subsequent template removal using an eluent created well-defined recognition cavities on the MOF surface. For comparison， non-imprinted polymers （CoNi-MOF-NIPs） were prepared following identical procedures without BPA addition. Critical synthesis parameters were systematically optimized through comprehensive experiments. The mass ratio of functional monomer to template molecule was determined to be optimal at 5∶4 （DA∶BPA）， while the polymerization duration and adsorption pH were optimized to 5 h and 4.0， respectively. Material characterization revealed crucial structural features： scanning electron microscopy （SEM） images confirmed the preservation of nanoflower-like morphology with hierarchical structures， providing abundant adsorption sites， while Fourier-transform infrared spectroscopy （FT-IR） demonstrated successful imprinting through characteristic peak shifts-notably the O-H stretching vibration migration from 3 443 cm⁻¹ to 3 752 cm⁻¹ and aromatic C=C bending transition from 1 633 cm⁻¹ to 1 457 cm⁻¹. Adsorption performance evaluation demonstrated remarkable efficiency across multiple aspects. Kinetic studies revealed rapid uptake conforming to pseudo-second-order behavior （*R*²=0.987 9 for CoNi-MOF-MIPs and *R*²=0.976 8 for CoNi-MOF-NIPs）， reaching equilibrium within 30 min and suggesting that the adsorption process was controlled by the availability of binding sites rather than diffusion limitations. Isotherm analysis showed excellent agreement with the Langmuir model， indicating monolayer adsorption on homogeneous surfaces with a maximum capacity of 39.29 mg/g and an impressive imprinting factor of 3.48. Competitive adsorption experiments against structural analogs （diphenolic acid and phenol） demonstrated exceptional selectivity， with selectivity factors of 5.07 and 7.35 respectively， confirming the material’s ability to specifically recognize BPA in complex matrices. The CoNi-MOF-MIPs maintained 93.2% of their initial adsorption capacity after six consecutive adsorption-desorption cycles， demonstrating excellent reusability. When coupled with high performance liquid chromatography （HPLC）， the developed analytical method exhibited a wide linear detection range （0.17-40 μg/mL， *R*²=0.997 4） and low detection limit （0.05 μg/mL）. Practical application to environmental water samples spiked at three concentration levels （10， 20， and 30 μg/mL） achieved satisfactory recoveries of 80.3%-91.7% with excellent reproducibility （relative standard deviation<1.8%）. The CoNi-MOF-MIPs material developed in this study combines the exceptional adsorption capacity of bimetallic MOFs with the molecular recognition precision of imprinted polymers. This innovative material demonstrates outstanding performance in both BPA enrichment and detection from environmental water samples， showing great promise for practical applications in water treatment and environmental monitoring.

双酚A（4，4′-二羟基二苯基丙烷，BPA）作为全球年产量超800万吨的高危内分泌干扰物，其化学稳定性与双酚基结构使其成为聚碳酸酯塑料（占全球塑料产量15%）和环氧树脂（食品罐涂层主要成分）的核心原料^［1，2］^。然而，这类聚合物材料在食品接触场景（如婴儿奶瓶、罐头内衬）中持续释放游离BPA，经迁移-溶解-扩散多级传递进入食品基质及环境水^［3］^。研究^［4］^表明，0.1 μg/L的BPA暴露即可通过雌激素受体*α*（ER*α*）介导途径干扰哺乳动物生殖发育，并诱导海马神经元Tau蛋白异常磷酸化（*p<*0.01），导致阿尔茨海默病相关生物标志物升高35%~40%。为此，中国（GB 9685-2016）、欧盟（No 10/2011）等50余国家及组织将食品接触材料中的BPA迁移限值严控至0.05 mg/kg，世界卫生组织更将环境水体的BPA安全阈值设定为0.1 μg/L^［5］^。

目前，针对BPA的检测方法已有报道^［6，7］^，高效液相色谱法（HPLC）因其灵敏度高、分析时间短、试剂消耗量小等特点，广泛用于环境水样中BPA含量的测定。分子印迹聚合物（MIPs）凭借“锁-钥”识别机制，适用于对BPA的特异性检测^［8，9］^。然而，现有BPA-MIPs材料仍存在吸附容量低和传质阻力高等方面的局限，难以满足复杂基质中BPA的高效富集需求。针对这一挑战，本研究提出“双金属有机框架协同-表面印迹”策略：通过Co^2+^/Ni^2+^配位构建双金属有机框架（CoNi-MOF），其三维多孔结构（孔径3.8 nm）和双活性位点可为模板分子预组装提供高密度锚定位点；采用盐酸多巴胺（DA）原位氧化自聚合在MOF表面构筑纳米级印迹层（厚度（50±5）nm），通过*π-π*堆积和氢键协同作用锁定BPA空间构型。这种核壳结构设计使吸附位点暴露度提升70%，结合双金属Lewis酸位点对BPA酚羟基的特异性捕获，成功实现了吸附容量与选择性的协同提高。本研究为复杂基质中痕量BPA的精准检测提供了创新解决方案。

## 1 实验部分

### 1.1 仪器与试剂

LC-20A HPLC系统包括紫外检测器（SPD-20A）、自动进样器（SIL-20A）、液体输送泵（LC-20AT）、柱温箱（CTO-20A）和Labsolutions工作站（Shimadzu，日本岛津公司）；JSM 6700-F型扫描电子显微镜（SEM）（日本JEOL公司）；Tensor Ⅱ型傅里叶变换红外光谱仪（FT-IR，德国布鲁克公司）；Plus-E2超纯水机（南京易普易达科技发展有限公司）；PHSF-3F pH计（上海精密科学仪器公司）；DOA-P504-BN型泵（美国GAST公司）。

DA（98%）、三（羟甲基）氨基甲烷（Tris）、2-甲基咪唑（2-MeIM）、BPA（98%）、二酚酸（DPA，98%）、苯酚（BP，99%）、六水合硝酸钴（Co（NO_3_）_2_·6H_2_O，99%）、六水合硝酸镍（Ni（NO_3_）_2_·6H_2_O，99%）购自上海麦克林公司。氢氧化钠（NaOH，98%）、磷酸（H_3_PO_4_，85%）、乙酸（HAc，99.5%）、乙醇（EtOH，98%）、甲醇（MeOH，98%）和盐酸（HCl，37%）购自上海化学试剂公司。以MeOH为溶剂，制备质量浓度为1 000 mg/L的BPA、DPA、BP原液，并在4 ℃下保存。以Na_2_HPO_4_和NaH_2_PO_4_为原料，通过加入NaOH或H_3_PO_4_来调节溶液pH值，制备磷酸盐缓冲溶液（PBS）。实际江水样品来自哈尔滨松花江，湖水样品取自哈尔滨师范大学梦溪湖。

### 1.2 双金属MOF的制备

采用简单、快速的方法在室温下合成了CoNi-MOF。具体步骤如下：12 mmol Co（NO_3_）_2_·6H_2_O和4 mmol Ni（NO_3_）_2_·6H_2_O溶于120 mL MeOH中，12 mmol 2-MeIM溶于40 mL MeOH中。将两种溶液快速混合，在室温下搅拌6 h，用MeOH反复洗涤，10 000 r/min离心。最后，在50 ℃真空干燥12 h制备CoNi-MOF。

### 1.3 CoNi-MOF-MIPs的制备

CoNi-MOF-MIPs制备过程如图1所示。首先，将CoNi-MOF作为基质，以DA作为功能单体、BPA为模板分子，基于DA在弱碱性条件下自聚合的原理，通过氢键和*π*-*π*相互作用将模板BPA固定在CoNi-MOF表面。最后，利用洗脱液破坏BPA和DA的相互作用，从而制得CoNi-MOF-MIPs。

**图1:**
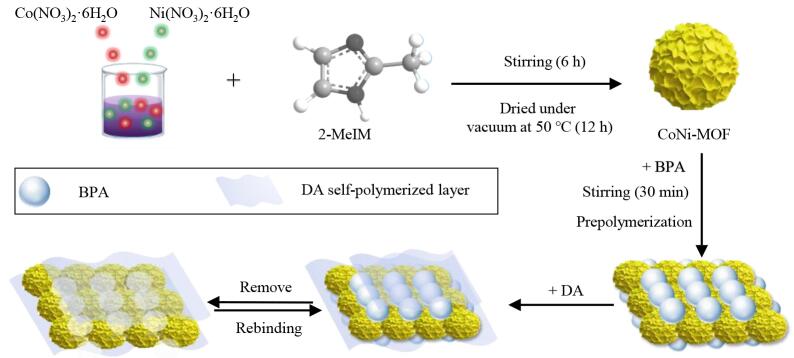
CoNi-MOF-MIPs的制备过程

具体制备方法如下：首先，在50 mL的烧杯中加入50 mg CoNi-MOF、20 mg BPA、20 mL Tris-HCl（pH 8.5）缓冲溶液，室温搅拌30 min预聚合。随后，向上述烧杯中加入25 mg DA，混合物在室温下搅拌5 h，离心（10 000 r/min）5 min，收集。最后，采用MeOH-HAc（9∶1，体积比）洗脱去除模板分子BPA，超声清洗6次，每次30 min，离心，更换清洗液，直至上清液中检测不到BPA，然后将溶液离心（10 000 r/min）5 min，离心得到的固体物质在50 ℃下烘干12 h，制备得到CoNi-MOF-MIPs。非表面分子印迹聚合物（CoNi-MOF-NIPs）的制备除不添加模板分子BPA以外，其余步骤同上。

### 1.4 材料的吸附性能考察

#### 1.4.1 动力学吸附性能

取2 mg CoNi-MOF-MIPs/CoNi-MOF-NIPs加入2 mL 200 mg/L BPA溶液中，室温振荡（220 r/min）5、10、15、20、25、30、35、40 min，离心5 min（10 000 r/min），将吸附材料与溶液分离，用HPLC检测上清液中BPA的含量。平衡吸附量*Q*（mg/g）利用公式（1）进行计算。


*Q*=（*C*_0_-*C*_e_）*V*/*W* （1）

式中，*C*_0_和*C*_e_（mg/L）分别为BPA的初始浓度和平衡浓度，*V*（mL）和*W*（mg）分别表示BPA溶液的体积和CoNi-MOF-MIPs（CoNi-MOF-NIPs）吸附剂的质量。

#### 1.4.2 等温吸附性能

取2 mg CoNi-MOF-MIPs/CoNi-MOF-NIPs分别放入2 mL质量浓度为50、100、150、200、250、300 mg/L的BPA溶液中，室温振荡（220 r/min）60 min后，离心5 min（10 000 r/min），将吸附材料与溶液分离，用HPLC测定上清液中BPA的含量，从而计算吸附量。

#### 1.4.3 选择性与竞争性吸附性能

为了探索CoNi-MOF-MIPs/CoNi-MOF-NIPs对BPA的选择性识别能力。取2 mg CoNi-MOF-MIPs/CoNi-MOF-NIPs分别放入2 mL质量浓度均为100 mg/L的BPA、DPA、BP混合溶液中，室温振荡（220 r/min）60 min后，离心5 min（10 000 r/min），将吸附材料与溶液分离，用HPLC测定上清液中BPA、DPA、BP的含量。

将干扰物质DPA与BP的浓度增大10倍，BPA浓度保持不变。取2 mg CoNi-MOF-MIPs/CoNi-MOF-NIPs分别放入2 mL三者混合溶液中，其余操作与选择性操作相同。印迹因子（IF）和选择性因子（SC）用来评估印迹材料对模板分子的选择性性能，其计算公式如下。

IF=*Q*_MIP_/*Q*_NIP_ （2）

SC=IF_TEM_/IF_COM _ （3）

式中，*Q*_MIP_与*Q*_NIP_分别代表CoNi-MOF-MIPs和CoNi-MOF-NIPs对模板分子BPA的吸附量；IF_TEM_和IF_COM_分别表示材料对BPA和其他竞争物的印迹因子。

### 1.5 色谱条件

Syncronis C18色谱柱（250 mm×4.6 mm，5 μm，美国赛默飞世尔科技有限公司）；柱温30 ℃，流速1.0 mL/min，进样量10 μL，检测波长224 nm，流动相为甲醇-水（70∶30，体积比）。使用前，流动相需经过0.45 μm滤膜过滤，然后脱气20 min。所有HPLC进样溶液均需过0.45 µm微孔滤膜过滤。

## 2 结果与讨论

### 2.1 聚合条件与吸附条件的考察

为获得更多的特异性识别位点和良好的吸附效果，对功能单体DA的用量、聚合时间以及吸附pH值进行考察，从而获得最佳的印迹效果。

固定50 mg CoNi-MOF底物、20 mg模板分子BPA不变，分别选取用量5、10、15、20、25、30 mg DA进行考察（图2a）。结果表明，随着DA用量的增加，吸附量和印迹因子先增加后略有减小；在其用量增加至25 mg之前，印迹层的厚度不断增加致使被包裹的BPA分子增多，洗脱后留下的特异性吸附位点数量增加，从而导致吸附量不断增加；在DA用量为25 mg时*Q*和IF最大；随着DA用量继续增加，*Q*和IF逐渐下降，分析可能是印迹层过厚使得包覆在内部的模板分子BPA不易完全洗脱，从而造成模板泄露并阻碍吸附传质过程或多余的DA发生自聚合。因此确定DA的最佳用量为25 mg。

**图2:**
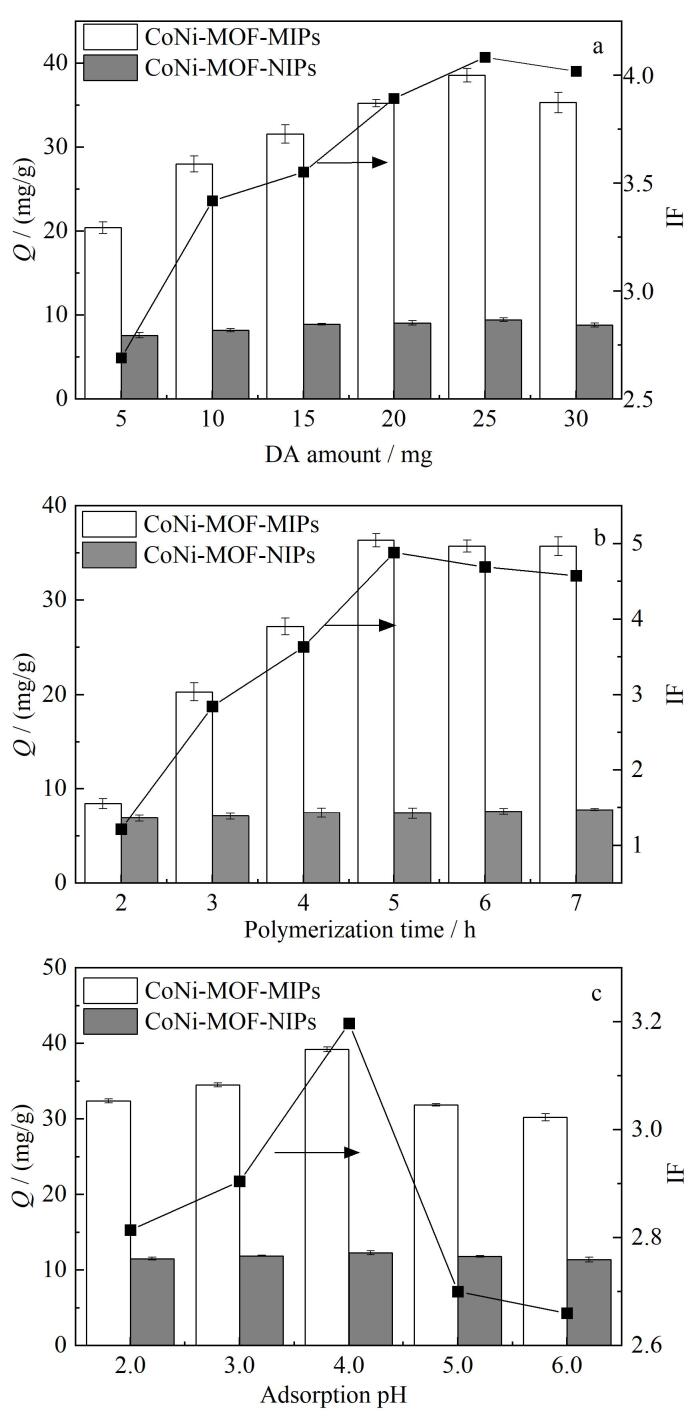
（a）DA用量、（b）聚合时间及（c）吸附pH对CoNi-MOF-MIPs/CoNi-MOF-NIPs吸附BPA效果的影响（*n*=3）

再固定DA用量为25 mg，选取聚合时间2、3、4、5、6、7 h进行考察（图2b）。结果表明，当聚合时间为5 h时，CoNi-MOF-MIPs显示出最高的吸附量和印迹因子，可能是在此时间下底物与模板分子结合形成的印迹位点数量以及印迹层厚度最合适；当聚合反应时间大于5 h时，*Q*和IF略有减小，主要是因为聚合时间过长导致包覆过深，模板分子不易洗脱且吸附过程中模板分子不易到达。由实验结果确定，最佳聚合时间为5 h。

在确定DA用量为25 mg、最佳聚合时间为5 h的前提下，为了获得更好的吸附效果，继续优化了吸附pH，取2 mg CoNi-MOF-MIPs，加入2 mL pH分别为2.0、3.0、4.0、5.0、6.0的100 mg/L的BPA溶液，在220 r/min转速的振荡器中室温振荡30 min后，离心5 min（10 000 r/min），将吸附材料与溶液分离，用HPLC测定上清液中BPA的浓度，从而计算出吸附量（图2c）。结果表明，当pH为4.0时，吸附效果最好。

### 2.2 材料表征

#### 2.2.1 扫描电镜分析

图3a、3b分别为CoNi-MOF和CoNi-MOF@BPA@DA的SEM图。可以看出CoNi-MOF是花瓣状结构且表面褶皱，具有较大的比表面积，该结构可以提供更多的吸附位点，经过DA包覆后，明显观察到印迹层的存在，同时也可以看到MOF的褶皱状态。

**图3:**
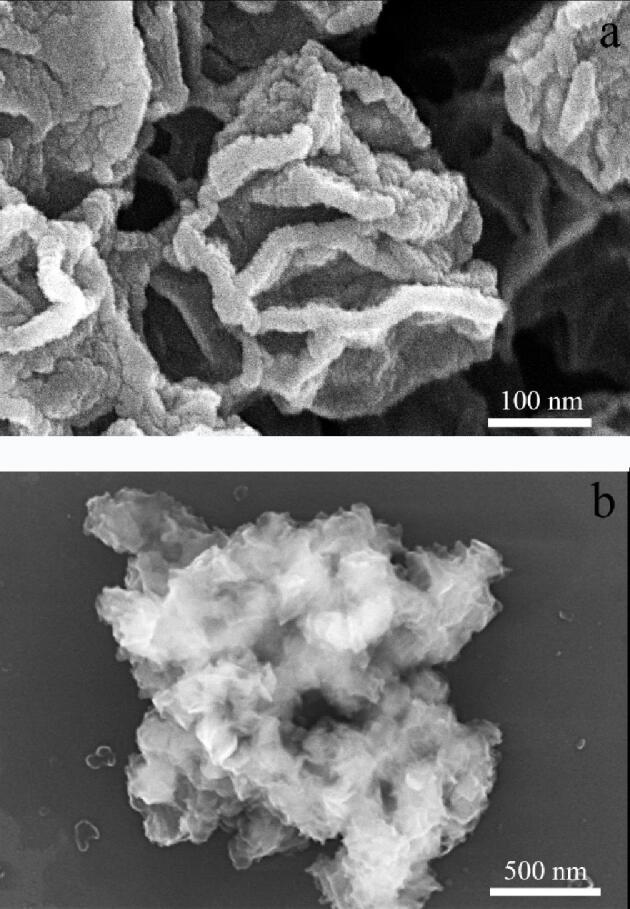
（a）CoNi-MOF和（b）CoNi-MOF@BPA@DA的扫描电镜图

#### 2.2.2 红外光谱分析

通过FT-IR对所制备的CoNi-MOF和CoNi-MOF@BPA@DA的化学键和官能团进行了表征，如图4所示，在CoNi-MOF FT-IR中，3 443 cm^-1^处的宽峰对应于羟基的伸缩振动，这可能是由配体中的羟基或样品干燥不完全吸附的水分子造成的，1 633 cm^-1^、2 938 cm^-1^处的吸收峰是由2-MeIM中的C=N和C-H伸缩振动引起的，1 385 cm^-1^和1 043 cm^-1^处的吸收峰分别代表C-O键和环状结构中的C-C键的伸缩振动，660 cm^-1^处的吸收峰反映金属-氧键的伸缩振动，来自Co或Ni与氧的配位。在CoNi-MOF@BPA@DA的FT-IR中，除与CoNi-MOF相似的特征峰以外，由于BPA和DA中含有较多酚羟基使对应于羟基伸缩振动的吸收峰发生红移，于3 752 cm^-1^处出现，在1 457 cm^-1^处特征峰表示苯环结构C=C的伸缩振动，3 012 cm^-1^处的吸收峰对应于芳香环中C-H伸缩振动，962 cm^-1^处的吸收峰对应于芳香环C-H的面外弯曲振动；在CoNi-MOF中，1 385 cm^-1^处产生的C-O的伸缩振动，而CoNi-MOF@BPA@DA在1 385 cm^-1^处吸收峰消失，表明CoNi-MOF@BPA@DA成功制备。

**图4:**
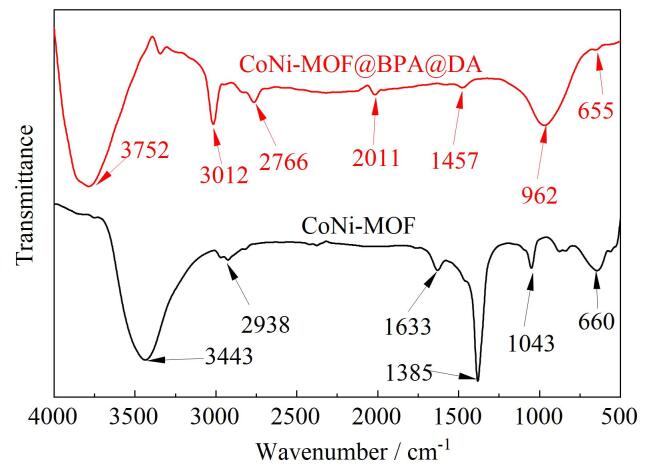
（a）CoNi-MOF和（b）CoNi-MOF@BPA@DA的红外光谱图

### 2.3 材料吸附性能考察

#### 2.3.1 动力学和等温吸附性能考察

对CoNi-MOF-MIPs/CoNi-MOF-NIPs进行5~40 min的动力学吸附实验，结果（图5a）表明吸附时间增加后，吸附量也随之增加，这是因为随着吸附时间的增加，CoNi-MOF-MIPs和CoNi-MOF-NIPs上的印迹识别位点逐渐与模板分子BPA结合；当吸附时间达30 min时，CoNi-MOF-MIPs/CoNi-MOF-NIPs的吸附量达到最大且几乎不再变化，表明材料上吸附位点与模板分子结合达到动态平衡。

**图5:**
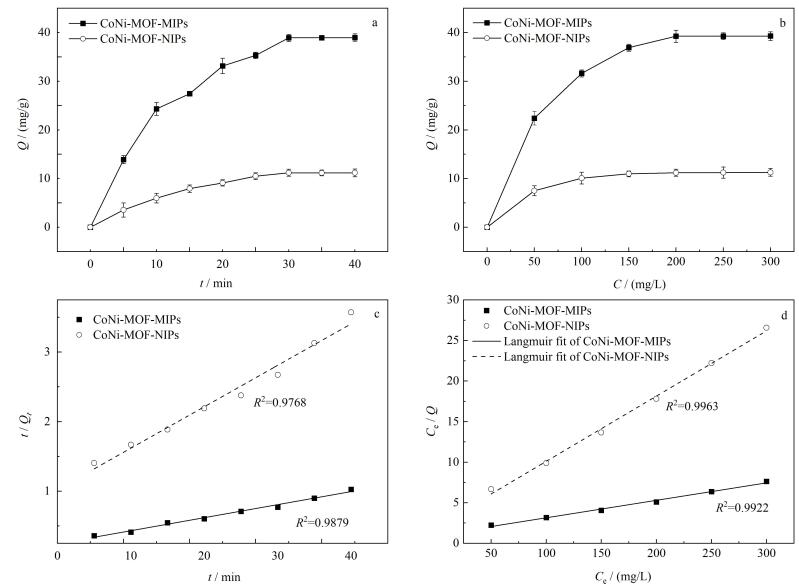
（a）动力学吸附图、（b）等温吸附、（c）拟二级动力学拟合曲线和（d）热力学Langmuir模型拟合曲线（*n*=3）

将CoNi-MOF-MIPs和CoNi-MOF-NIPs分散在50~300 mg/L的BPA溶液中，等温吸附实验结果（图5b）表明，当BPA质量浓度小于200 mg/L时，CoNi-MOF-MIPs的吸附量随着浓度的增大而增大，当BPA的质量浓度达到200 mg/L时，其吸附量达到最大（39.29 mg/g），继续增大BPA的质量浓度，吸附量几乎不变，这是因为在此浓度下CoNi-MOF-MIPs中特异性和非特异性识别位点与模板分子结合均已达平衡；而仅有非特异性识别位点的CoNi-MOF-NIPs的吸附量在BPA质量浓度小于150 mg/L时随着浓度的增大而增大，在浓度为150 mg/L时吸附达到平衡，其吸附量为11.29 mg/g，约为CoNi-MOF-MIPs的1/3。

采用拟一级和拟二级动力学模型拟合动力学数据，拟一级的线性相关系数（*R* ^2^）较小，偏差较大，拟二级动力学模型（图5c）更符合实验数据（*R* ^2^=0.987 9），这表示CoNi-MOF-MIPs和CoNi-MOF-NIPs吸附过程可能受位点数量控制，BPA需与空腔的氢键、*π*-*π*堆积位点按1∶1比例结合且吸附速率受限于空腔位点的占据速度，而非扩散过程。

为了进一步探究等温吸附过程，选择Langmuir模型和Freundlich模型对等温数据拟合，拟合结果表明，CoNi-MOF-MIPs（*R* ^2^=0.992 2）和CoNi-MOF-NIPs（*R* ^2^=0.996 3）对BPA的吸附行为更符合Langmuir模型（图5d），说明其对BPA的吸附可能是单层吸附，主要因为CoNi-MOF作为基底提供了均质孔道，其规则结构确保了吸附位点的能量一致性。同时，对于CoNi-MOF-MIPs来说，DA自聚合包裹模板分子BPA形成的印迹空腔，通过几何空间的匹配和多重氢键互补（酚羟基与PDA的邻苯二酚基团、氨基形成多重氢键），实现了对BPA的单层特异性物理吸附。

#### 2.3.2 选择性与竞争性吸附性能考察

本研究选择具有代表性的两种结构类似物DPA和BP来评估CoNi-MOF-MIPs和CoNi-MOF-NIPs对BPA的选择性识别能力（图6a）。结果表明，相同浓度下，CoNi-MOF-MIPs对BPA的*Q*和IF均远大于其他竞争物，且对DPA、BP的SC均较大，分别为5.75、8.68，证明CoNi-MOF-MIPs对BPA为特异性吸附，且选择性较好；而CoNi-MOF-MIPs和CoNi-MOF-NIPs对DPA、BP的吸附量均较小，为非特异性吸附。

**图6:**
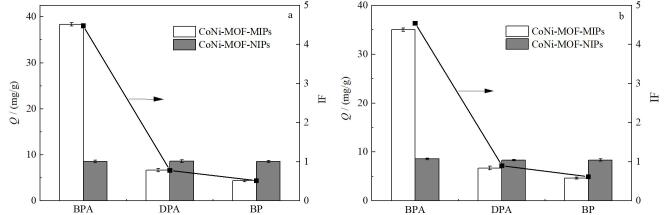
CoNi-MOF-MIPs和CoNi-MOF-NIPs对BPA及两种竞争物的（a）选择性与（b）竞争性吸附考察（*n*=3）

如图6b所示，BPA浓度不变，而其他竞争物的浓度扩大10倍后，CoNi-MOF-MIPs对BPA的吸附量虽略有下降但仍大于其他两种竞争物，且对DPA、BP的SC（5.07、7.35）仍较大，几乎无减少，表明CoNi-MOF-MIPs对BPA具有良好的竞争性；而CoNi-MOF-NIPs对3种物质的吸附量仍几乎相同。

#### 2.3.3 重复利用性能考察

采用同一CoNi-MOF-MIPs和CoNi-MOF-NIPs进行6次吸附-解吸附实验来考察材料的重复利用性能，实验结果如图7所示，随着材料吸附-解吸附次数增加，有一部分印迹位点因被模板分子BPA堵塞而难以洗脱，导致材料对BPA的吸附量略有减小。当重复使用次数达到6次时，CoNi-MOF-MIPs的吸附量仍然可以达到第一次吸附量的93.2%，证明该材料对BPA的吸附较稳定且具有良好的重复利用性。而CoNi-MOF-NIPs对BPA的吸附几乎未受洗脱次数影响。

**图7:**
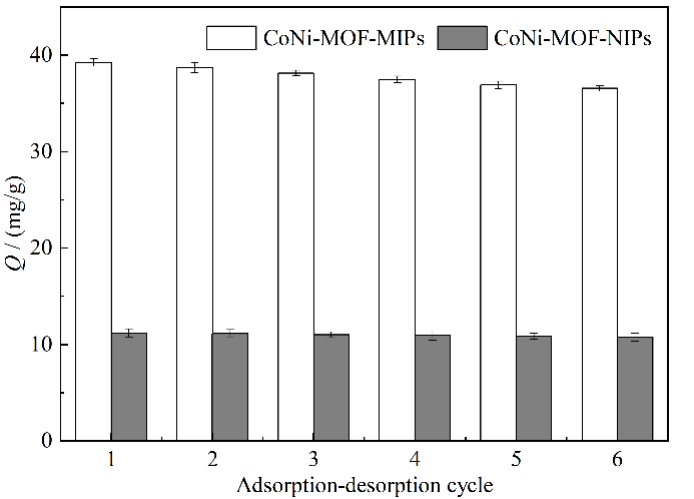
CoNi-MOF-MIPs和CoNi-MOF-NIPs的重复使用性（*n*=3）

#### 2.3.4 方法评估

本工作建立了一种以CoNi-MOF-MIPs作为固相萃取吸附剂结合HPLC检测环境水样中BPA的方法，BPA质量浓度在0.17~40 µg/mL的线性范围内具有良好的线性关系，*R* ^2^为0.997 4。分别以3倍信噪比和10倍信噪比确定检出限（LOD）为0.05 µg/mL，定量限（LOQ）为0.17 µg/mL。

#### 2.3.5 实际样品分析

本研究将制备的CoNi-MOF-MIPs作为吸附材料，与HPLC结合，用于检测环境水样中的BPA，对加标10、20和30 μg/mL BPA的江水和湖水样品进行分析，3个水平下加标回收率实验结果（表1）显示材料对加标的湖水和河水中BPA的回收率为80.3%~91.7%，RSD为0.6%~1.7%，表明该方法准确度较高且灵敏度较好。同时，该方法回收率和精密度良好，能够满足环境样品中BPA残留的定性和定量分析。

**表1:** 环境水样中BPA在3个水平下的加标回收率（*n*=3）

Sample	Spiked/（µg/mL）	Detected/（µg/mL）	Recovery/%	RSD/%
Lake water	10	9.17	91.7	0.6
	20	17.95	89.7	0.8
	30	26.45	88.2	1.3
River water	10	8.03	80.3	0.8
	20	16.47	82.3	0.9
	30	24.82	82.7	1.7

此外，由图8可以看出，加标湖水中虽然检测到BPA（图8b），但有其他杂峰出现，表明湖水为复杂基质。CoNi-MOF-MIPs吸附加标湖水样品后利用洗脱液将BPA洗脱并烘干，将剩余物溶解，经分析，在约5.8 min时出现尖锐清晰的BPA吸收峰（图8c），这与BPA标准品的保留时间相吻合（图8a）。这些结果表明，CoNi-MOF-MIPs作为固相萃取吸附剂结合HPLC，实现了对环境水样中BPA的高效吸附、富集与检测。

**图 8:**
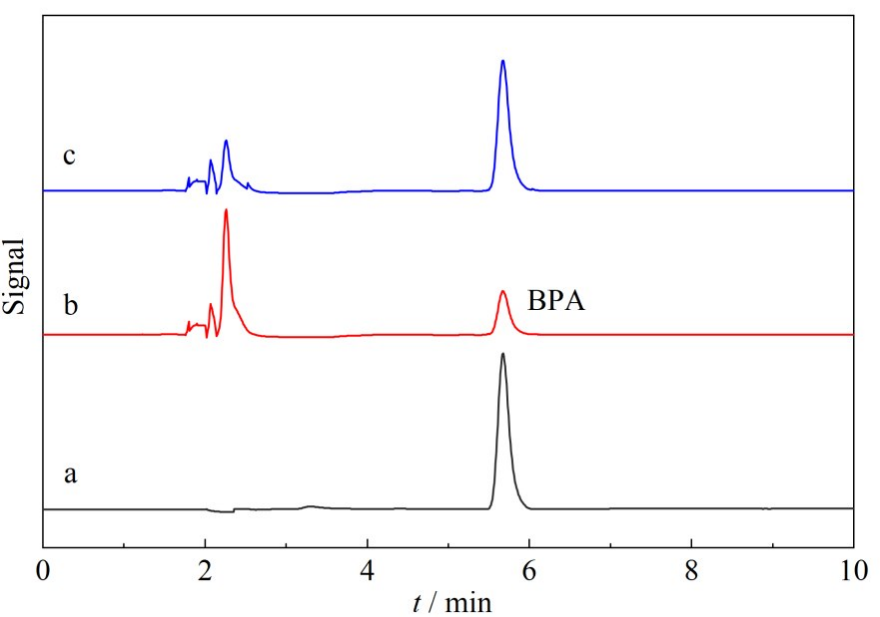
（a）BPA标准溶液和加标（10 μg/mL）湖水样品经CoNi-MOF-MIPs（b）富集前、（c）富集后的色谱图

### 2.4 与其他吸附剂的比较

将本工作所制备的CoNi-MOF-MIPs与其他5种BPA吸附材料的相关参数进行了比较^［10-14］^，对比结果如表2所示。与其他5种材料相比，虽然本研究的LOD、LOQ值不是最低的，但吸附能力较强，最大吸附量达39.29 mg/g，较rFe_3_O_4_@ZIF-8@MIP高约4倍，IF较高，RSD较低。由此可见，本工作所制备的CoNi-MOF-MIPs能准确检测出实际样品中的BPA，可作为未来吸附BPA的重要材料之一。

**表2:** 不同吸附剂对BPA分析性能的比较

Adsorbents	*Q/*（mg/g）	IF	LOD	LOQ	RSD/%	Ref.
rFe_3_O_4_@ZIF-8@MIP^a^	10.10	2.72	0.1 µg/L	—	3.6	［^10^］
VTTS-MGO@mSiO_2_@MIPs^b^	16.81	4.18	0.010 µg/L	2.68 µg/L	5.01-5.73	［^11^］
DES-MIP^c^	17.72	2.02	—	—	—	［^12^］
S-MIPs^d^	36.00	1.76	0.0017 mg/L	—	—	［^13^］
E-TBBPA-MIM^e^	—	6.31	—	—	—	［^14^］
CoNi-MOF-MIPs	39.29	3.48	0.05 µg/mL	0.17 µg/mL	0.60-1.7	this work

a. magnetic metal-organic frameworks molecularly imprinted nanoparticles； b. mesoporous silica coated magnetic graphene oxide multi-templates molecularly imprinted polymer； c. deep eutectic solvent molecularly imprinted polymer； d. solvent-responsive molecularly imprinted polymers； e. elute TBBPA molecularly imprinted nanoparticles.

## 3 结论

本研究以纳米花状的CoNi-MOF为基底，BPA为模板分子，DA为功能单体，通过DA与金属配位协同作用构建了具有高特异性识别位点和高吸附量的表面分子印迹聚合物（CoNi-MOF-MIPs）。本研究所制备的新型印迹材料具有较快的吸附平衡时间、较大的吸附量、较高的选择性、优异的竞争性和极好的重复利用性。此外，本研究建立的以CoNi-MOF-MIPs作为吸附剂的HPLC检测方法应用于环境水样中BPA的选择性检测与富集，相对标准偏差较小，数据检测结果稳定，为复杂基质中痕量BPA的高效富集与检测提供了兼具高吸附量与特异识别性能的材料体系，该检测方法在环境检测领域具有潜在的应用价值。
